# 40 Years of History of the Journal *Investigación y Educación en Enfermería*. Advancing in Knowledge*

**DOI:** 10.17533/udea.iee.v41n3e14

**Published:** 2023-10-31

**Authors:** Jaime Horacio Toro-Ocampo

**Affiliations:** 1 Nurse, Magíster. Profesor Associate Professor at Facultad de Enfermería de la Universidad de Antioquia, Colombia. Email: horacio.toro@udea.edu.co Universidad de Antioquia Facultad de Enfermería Universidad de Antioquia Colombia horacio.toro@udea.edu.co

Forty years ago, when the journal *Investigación y Educación en Enfermería* was founded, it had the vision of being the main means of expression of the Faculty of Nursing at Universidad de Antioquia. Within this projection, from its beginnings, it sought to be the channel for the exchange of knowledge by nursing professionals in the country and in Latin America. To celebrate these anniversaries, a compilation was made of the most important information in the history of the journal, inviting us to reflect on the many achievements it has granted to our Faculty; also, recognition is given to everyone who has contributed to the journal and to the profession.

## First decade

The first issue of the journal was launched in September 1983. At the time, the Editorial Board was made up of Consuelo Castrillón Agudelo, who was its first director, and by Amparo Zapata Villa, Jaime Mercado and Orlando Sáenz Zapata, who were the first participants of the Editorial Board. Dean Liria Pérez Peláez was in charge of launching the journal. The research lines proposed for the publication of articles were: epidemiology, education, health services administration, social medicine, and basic sciences. In this first edition, the journal’s content was composed by four research articles, three on education, a commented publication and the dissemination of the event “Nursing Research Colloquium”; this first issue had a cost of $200 Colombian pesos. In 1984, 16 standards were regulated for article publication, and the International Standard Serial Number (ISSN) 0120-5007 was acquired, which is the international identification number assigned to periodical publications. The back cover of the second issue mentioned the journal's distribution committee made up of Beatriz Zuluaga, Constanza Forero, and Clara Sánchez. The front cover design and diagramming was carried out by publicist Mario Peláez. This year, 19 articles were published - distributed in two issues. 

During the following years, under the direction of **María Consuelo Castrillón Agudelo** (1983 - 1987), the following progress was made: inventory of research conducted by nurses or with some participation in the department of Antioquia. To have a distinctive cover, color played an important role in identifying the journal and giving it a characteristic seal. From this perspective, the green journal was identified as the first issue, the fuchsia and blue journal were the following issues published in 1984, the yellow journal of 1985 focused on the health of the elderly, the publication for September 1989 was the black journal; this color was selected because it corresponded to a dark period for the University, as it was facing the murder of academics and students at the time.

Another relevant aspect during this decade was the leadership role played by **Beatriz Zuluaga Ángel** (1983 - 1990), as a member of the physical and personal distribution committee of the journal's copies. For this, contacts were established with libraries from the faculties of medicine, nutrition, and bacteriology, in addition to distributing in the Noel Clinic, the Social Security, León XIII Clinic, Hospital Universitario San Vicente de Paul University Hospital, among others. Advertising began to be sold to laboratories for financing and distribution was expanded nationwide, for which an agreement was reached with the Postal Administration to deliver to different parts of the country.

The journal’s second director was **Martha Lucía Palacio Campuzano** (1985- 1988). In this period, submission to BIREME stands out, in the National Data Bank and in the National Information Center of Medical Sciences in Cuba. 

Under the direction of **Silvia Orrego Sierra** (1988 - 1990), the first five years of the journal were celebrated and linked to the celebration of the 40 years of the Faculty of Nursing. In this period, a special section was made on health care during disaster situations. 

From the period (1991-1992) under the direction by **Martha Lucia Toro Restrepo**; representative paintings on health were included on the cover (1991) and 19 criteria to be taken into account for the publication of scientific articles were added to the authors’ instructions. In this period, use of keywords was included, allowing for easier search of topics of the articles. Advertising guidelines were expanded with Nestlé, Cisne Blanco, and Lumar. Additionally, shape changes were made to the journal’s cover and the back cover in color was included as of then.

## Second decade

Under the direction by **Luz Ángela Ramírez Jaramillo** (1992 - 1998) ten years of the journal were celebrated. Other challenges included: from 1996, abstracts in English of all articles; participation in the PUBLINDEX call by COLCIENCIAS (today MinCiencias) which resulted in classification in category “A”, which is why it received an important economic stimulus. For the following year, the Consulting Council was created for the first time with international expert participation; color images began to be published in the journal’s articles, and the size and presentation format were also changed. 

With guidance by **Amparo Zapata Villa** (1998 - 2001), it was proposed that each publication would have a specific theme for publication and together with the Editorial Board, the themes for each annual edition were defined, thus: disease prevention and health promotion (1998), nursing in Colombia and the dimension of care (1999), tribute to Virginia Henderson and Dorothea Orem (2000), health emergencies (2001), and women's health (2002). In 2000, with the tribute to theorists, the journal’s format changed. In this direction, it was important to enter the digital era with the development of the first website, making it easier to read articles on the Internet.

Under the direction by **Clara Inés Giraldo Molina** (2001-2004), the journal worked on increasing the publication’s scientific quality with international participation of authors, referees, and members of the Editorial Board, the foregoing led to indexing the journal in Cuiden Spain and BIREME Brazil. Also, to comply with this objective, the Authors’ Instructions were expanded in detail. The last challenge for this direction was the celebration of the journal’s 20 years.

## Third decade

During the period of the direction by **Bertha Ligia Díez Mejía** (2004 - 2008), national and international recognition was achieved. The journal, classified by COLCIENCIAS in category “B”, managed to increase to “A2”. With compliance with periodicity, a pleasant and easy-to-read format, participation from a group of referees with extensive experience and recognized prestige within the profession and in the area of health, the journal began digital dissemination on the OJS platform with an important number and variety of articles from different countries. *Investigación y Educación en Enfermería* was indexed in SciELO, LILACS, Thomson Academic-OneFile, LATINDEX, DIALNET, Virtual Health Library and in the Ibero-American Council of Editors of Journals in Nursing -CIBERE-. Also, this administration decided to reduce circulation to 700 and later to 500 copies, taking into account that there was already a digital presentation, in addition to the printed one.

The direction by **María del Pilar Pastor Durango** (2008 - 2009) worked on enhancing the journal’s visibility and maintaining its classification in PUBLINDEX. Progress during this period focused on systematizing processes carried out in the journal and on constructing a methodological memory of its operation that facilitates induction of new individuals. The journal transitioned in cover design towards photographs that showed different facets of nursing care. The journal's website was updated and installed on the Toné server at Universidad de Antioquia, which had manuals for its installation, configuration, administration, and interaction with authors, reviewers, and editors. The subscriber database was updated and two management systems were developed to facilitate delivery processes (subscribers) and inventory management (DBJournal). In addition, construction of a database began to update the resumes of peer reviewers and authors.

## Fourth decade

The period of **María de los Ángeles Rodríguez Gázquez** as editor began prior to this decade (2009 - to date). During her management, the following advances have been made: since 2014, all articles have been published in English, which has increased reception of manuscripts from abroad, coming from four continents: America, Europe, Asia and Oceania. In 2019, after 36 years of being printed, the journal *Investigación y Educación en Enfermería* became an exclusively electronic publication. These are the challenges towards open science that have been advanced: the journal’s articles are available from volume 1 number 1 to the present in open access on the portal of journals of Universidad de Antioquia. Since 2017, all articles are identified with DOI and, as of 2020, authors’ ORCIDS are shown and the declaration was added of the use of the Creative Commons BY-NC-SA license, in addition to being indexed in the Directory of Open Access Journals (DOAJ). As for indexing in high-profile bibliographic bases, this has been the itinerary: Medline-PubMed (2014), Scopus (2015), PubMed Central (2020), Emerging Sources Citation Index by Web of Science (2021), Journal Citation Report of Web of Science (2022). With regard to the latter, an impact factor of 2 was obtained, which meant that *Investigación y Educación en Enfermería* is the nursing journal with the highest impact in Latin America.

**Table 1:** Numbers published by year of the journal *Investigación y Educación en Enfermería*

Year	Numbers published
1983	First and only number published in the year
1984 - 2009	Two numbers per year
2010 **-** to date	Three numbers per year
1993 and 2008	Special issue and Supplement

**Table 2:** Recount of the most relevant advances in the history of the journal *Investigación y Educación en Enfermería*

Year	Relevant data
1983	Creation of the journal Creation of the Editorial Board
1984	16 guidelines in the Authors’ Instructions First Director Formation of the Editorial Committee Formation of the Distribution Committee Assignment of ISSN: 0120- 5307
1985	Cover funded by ANEC
1986	Second dissemination of the event 5^th^ Conference on Social Medicine
1987	First publicity page by “Proleche” Black cover (Persecution against the University)
1988	Submission to: BIREME, National Data Bank of Colombia and National Information Center of Medical Sciences of Cuba
1989	First opinion article published Between each article, advertising from Nestlé, Cisne Blanco, Lumar, among others, is published
1990	Change of journal’s cover Back cover in color
1991	Back cover with paintings representative of health Authors’ Instructions include 19 guidelines for publication
1992	Authors’ Instructions include 21 guidelines for publication
1993	Celebration of 10 years of the journal Authors’ Instructions include 23 guidelines for publication
1994	The creation of the Master's Degree in Collective Health was publicized
1996	First article with abstract in English Authors’ Instructions include 20 guidelines for publication COLCIENCIAS classified the journal in category A Journal’s back cover includes figures from the Museum at Universidad de Antioquia
1997	Authors’ Instructions changes to a more-detailed and descriptive format The Advisory Council was created
1998	First article published with an image in color
1999	Started using publicity in color Digital publication begun in the Web site: http://caribe.udea.edu.co
2000	Change in the journal’s size and format
2001	Indications were attached for the creation of advertising guidelines Cover and back cover in color PUBLINDEX by COLCIENCIAS classified the journal in category C 1000 copies were printed
2002	First use of bar code
2003	New editorial news section Journal indexed in: Cuiden (Spain) and BIREME (Brazil) Celebration of 20 years of the journal
2004	Print run is reduced to 700 copies
2005	COLCIENCIAS classified the journal in category B Indexed in LILACS
2006	Print run is reduced to 500 copies
2007	Indexed in SciELO - Colombia Classification A2 in PUBLINDEX by COLCIENCIAS
2008	Indexed in: Thomson Academic-OneFile, LATINDEX, DIALNET, Biblioteca Virtual de la Salud (BVS) and in Consejo Iberoamericano de Editores de Revistas en Enfermería (CIBERE)
2010	Change of publication language from Spanish to English. All articles have abstracts in English, Spanish, and Portuguese Change in cover, back cover, and interior format Print run is reduced to 300 copies Periodicity increased to three numbers per year
2012	Indexed in: Directory of Open Access Journals (DOAJ),
2013	Print run is reduced to 300 copies
2014	Indexed in Medline Print run is reduced to 250 copies
2015	Indexed in Scopus and classified in quartile 3
2016	Change in cover, back cover, and interior format Print on recycled paper
2017	All articles are identified with DOI Journal is no longer printed and since then is exclusively electronic
2018	Submitted to Web of Science
2019	Submitted to PubMed Central
2020	The Journal is indexed in: PubMed Central Authors’ ORCIDs shown Declaration added of the use of the Creative Commons license
2021	Change of classification from 3^rd^ to 2^nd^ quartile in Scopus
2022	Indexed in Journal Citation Report by Web of Science, obtaining an impact factor of 2.0, which recognizes *Investigación y Educación en Enfermería* as the journal with the greatest impact on nursing in Latin America
2023	Celebration of the Journal’s 40 years since its creation

Note: this article gathered information from the collection of articles published in the Journal since its start until 2023, and extracted essential data from these articles, specially:

Díez-Mejía BL, Castrillón-Agudelo MC, Zuluaga-Ángel B, Palacio ML, Orrego-Sierra S, Toro-Restrepo ML, Ramírez-Jaramillo LA, Zapata-Villa A, Giraldo-Molina CI. Journal *Investigación y Educación en Enfermería*. An approach to its history. Invest. Educ. Enferm. 2008; 26(Supl): 16-41.

Presentation. Invest. Educ. Enferm. 1983; 1(1):5-8.

